# Complete genome sequence of *Treponema pallidum *ssp. *pallidum *strain SS14 determined with oligonucleotide arrays

**DOI:** 10.1186/1471-2180-8-76

**Published:** 2008-05-15

**Authors:** Petra Matějková, Michal Strouhal, David Šmajs, Steven J Norris, Timothy Palzkill, Joseph F Petrosino, Erica Sodergren, Jason E Norton, Jaz Singh, Todd A Richmond, Michael N Molla, Thomas J Albert, George M Weinstock

**Affiliations:** 1 Human Genome Sequencing Center, Baylor College of Medicine, One Baylor Plaza, Alkek N1619, Houston, TX 77030, USA; 2 Department of Biology, Faculty of Medicine, Masaryk University, Kamenice 5, Building A6, 625 00 Brno, Czech Republic; 3 Department of Pathology and Laboratory Medicine, University of Texas-Houston Medical School, 6431 Fannin Street, Houston, TX 77030, USA; 4 Department of Molecular Virology and Microbiology, Baylor College of Medicine, One Baylor Plaza, Houston, TX 77030, USA; 5 Roche NimbleGen, Inc., 500 S. Rosa Road, Madison, WI 53719, USA; 6 Department of Molecular and Human Genetics, Baylor College of Medicine, One Baylor Plaza, Houston, TX 77030, USA

## Abstract

**Background:**

Syphilis spirochete *Treponema pallidum *ssp. *pallidum *remains the enigmatic pathogen, since no virulence factors have been identified and the pathogenesis of the disease is poorly understood. Increasing rates of new syphilis cases per year have been observed recently.

**Results:**

The genome of the SS14 strain was sequenced to high accuracy by an oligonucleotide array strategy requiring hybridization to only three arrays (Comparative Genome Sequencing, CGS). Gaps in the resulting sequence were filled with targeted dideoxy-terminators (DDT) sequencing and the sequence was confirmed by whole genome fingerprinting (WGF). When compared to the Nichols strain, 327 single nucleotide substitutions (224 transitions, 103 transversions), 14 deletions, and 18 insertions were found. On the proteome level, the highest frequency of amino acid-altering substitution polymorphisms was in novel genes, while the lowest was in housekeeping genes, as expected by their evolutionary conservation. Evidence was also found for hypervariable regions and multiple regions showing intrastrain heterogeneity in the *T. pallidum *chromosome.

**Conclusion:**

The observed genetic changes do not have influence on the ability of *Treponema pallidum *to cause syphilitic infection, since both SS14 and Nichols are virulent in rabbit. However, this is the first assessment of the degree of variation between the two syphilis pathogens and paves the way for phylogenetic studies of this fascinating organism.

## Background

*Treponema pallidum *subspecies *pallidum *(TPA) is the causative agent of syphilis, a sexually transmitted disease affecting more than 12 million people worldwide each year [1]. After a period of decline in the 1990s, the number of reported cases of primary and secondary syphilis has been raising annually since 2000 in the United States [2]. Sequencing of the 1.14 Mbp genome of the Nichols strain of TPA in 1998 [3] greatly stimulated study of this unculturable pathogen. One important direction not yet developed is use of the Nichols sequence for comparative studies to determine variation between different syphilis isolates, how representative Nichols is of TPA, and the genetic differences between closely related treponemes causing different diseases (e.g. syphilis, yaws, bejel, pinta). To sample strains on a sufficient scale, rapid, inexpensive, and highly accurate sequencing methods are needed. Traditional whole genome shotgun sequencing methods using dideoxy-terminators (WGS-DDT) are relatively slow and costly to be applied to numerous samples. Here we sequence a treponemal genome by Comparative Genome Sequencing (CGS) [4], which provides an alternative to WGS-DDT sequencing of closely related genomes. CGS was previously used for mutation discovery in viruses [5], in mutagenized laboratory bacterial and fungal strains [4,6-9], in clinical isolates of bacteria [10,11], and for whole genome scale comparative studies [12-14].

The TPA isolate Street Strain 14 (SS14) was isolated in 1977 in Atlanta from a patient with secondary syphilis [15] who did not respond to erythromycin therapy that was used because of a penicillin allergy [16]. *In vitro *testing of SS14 revealed it to be less susceptible to a variety of antibiotics when compared to Nichols [16]. Nichols strain was isolated in 1912 in Washington, D.C. from cerebrospinal fluid of the patient with neurosyphilis [17]. Previous studies (D. Šmajs, G. M. Weinstock, unpublished results) showed SS14 had all genes of the Nichols genome as judged by hybridization to a microarray containing PCR products of all annotated Nichols open reading frames (ORFs) [18]. To compare these closely related, yet phenotypically distinct strains, we sequenced the SS14 genome by CGS.

## Results

### Identification of heterologous regions and sequence changes between Nichols and SS14 strains

In the first mapping stage of CGS, no regions with significantly stronger labeled SS14 DNA signals were observed, indicating no increase in gene copy number in the SS14 genome. Regions giving significantly weaker SS14 signals indicated 1731 candidate regions of variation encompassing 1 or more overlapping oligonucleotide targets. The sequencing data identified 213 SNPs in the SS14 genome. An additional 17 questionable SNPs were suggested in repeated sequences of the genome but did not score well in a SNP uniqueness algorithm [4], and thus could represent false positives due to cross hybridization with the other repeats. DDT sequencing of 12 such regions revealed 5 real SNPs, 6 false positives, and one position with 2 alleles within the SS14 population (intrastrain heterogeneity). Therefore these questionable SNPs were not included in the final sequence, unless they were verified by DDT sequencing (data not shown). An additional 62 positions out of the 213 SNPs identified by CGS were DDT sequenced. 60 SNPs were confirmed (Tables 1, 2, 3 last column) and 2 false positives were found.

**Table 1:** DDT sequencing of 38 hypervariable regions where SNPs could not be identified by CGS

Region no.	ORF^a^	Region size (nt)	Size of sequenced region (nt)	Left coordinate^a^	Right coordinate^a^	Newly found changes in the regions suggested by CGS	Newly found changes not suggested by mapping phase of CGS	Confirmation of SNPs identified by CGS in this region^b^
1	TP0012	37	390	12322	12711	3 nt deletion	-	-
2	TP0076	29	529	83788	84316	-	1 solitary SNP	-
3	TP0117	86	699	134808	135506	7 clustered SNPs	-	-
4	TP0117	86				3 clustered SNPs	-	-
5	TP0126	29	393	147948	148340	1 solitary SNP	-	-
6	upstream	29	460	149103	149562	2 clustered SNPs	-	-
	of TP0128					1 nt + 5 nt insertions		
7	TP0131	421	723	150925	151647	3 clustered SNPs, 1 solitary SNP	2 clustered SNPs	-
8	upstream of TP0136	29	404	156348	156751	64 nt deletion	-	-
9	TP0136	1087	1609	156752	158360	19 clustered SNPs	8 clustered SNPs	21 SNPs
						1 nt + 1 nt + 1 nt + 6 nt deletions	2 solitary SNPs	
10	TP0272	29	480	288647	289126	-	-	-
11	TP0304	37	466	318761	319226	3 nt deletion	-	-
12	TP0326	79	452	345605	346056	8 clustered SNPs	-	1 SNP
13	TP0352	29	465	376926	377390	-	-	-
14	TP0394	29	505	420353	420857	-	-	1 SNP
15	TP0431	29	465	458973	459437	-	-	1 SNP
16	TP0457	29	465	487935	488399	-	-	-
17	TP0484	29	468	514441	514908	-	-	-
18	TP0486	29	494	517297	517790	-	1 nt insertion, 1 nt deletion	-
19	TP0493	29	478	529146	529623	-	-	-
20	TP0515	44	506	555754	556259	3 clustered SNPs	-	4 SNPs
21	TP0544	29	611	585940	586550	6 nt insertion	-	-
22	TP0548	835	1189	591557	592745	22 clustered SNPs	2 clustered SNPs	5 SNPs
						3 nt + 4 nt + 5 nt insertions	1 solitary SNP	
23	TP0577	37	405	628247	628651	1 solitary SNP	-	-
24	TP0598	29	550	648851	649400	-	4 1 nt insertions	-
25	TP0620–TP0621	51	3469	670958	674426	-	4 clustered SNPs	-
26	TP0668	37	462	730080	730541	6 nt deletion	-	-
27	TP0699	51	469	766143	766611	1 solitary SNP	-	-
28	TP0785	29	438	851631	852068	-	-	-
29	TP0814	29	476	882990	883465	-	-	-
30	TP0865	29	480	943847	944326	3 nt insertion	-	1 SNP
31	TP0866	29	543	944677	945219	-	1 nt insertion	-
32	TP0868	29	454	947257	947710	7 nt deletion	-	-
33	TP0896–TP0898	667	3038	974053	977090	4 SNPs^c ^and 7 variable regions^d^		1 SNP
34	TP0898	27	416	978349	978764	-	-	-
35	TP0933	29	164	1014034	1014197	-	-	-
36	TP0973	44	396	1057660	1058055	1 solitary SNP	-	1 SNP (igr)
37	TP1030–TP1031	1507	402	1123660	1124061	18 clustered SNPs	1 nt insertion,	16 SNPs
			1775	1124256	1126030		1 solitary SNP	
38	TP1036	29	550	1132558	1133107	-	-	-

ORF – open reading frame; nt – nucleotide; SNP – single nucleotide polymorphism; igr – intergenic region; ^a^as described in [3]; ^b^SNPs identified using CGS in these regions were verified by DDT sequencing; ^c ^two SNPs represent the group of 17 SNPs in non-unique sites, originally excluded from list of total changes; ^d^identified variable regions in TP0897 were identical to the variable regions V1–V7 described previously [22–24].

**Table 2:** DDT sequencing of regions selected based on pilot SS14/Nichols comparison using microarray hybridization experiments

Region no.	ORF^a^	Size of sequenced region (nt)	Left coordinate^a^	Right coordinate^a^	Newly found changes	Confirmation of SNPs identified by CGS^b^
1	TP0017	848	18454	19301	-	-
2	TP0070	339	75493	75831	-	-
3	TP0094	1011	102879	103889	-	-
4	TP0123	1083	143207	144289	-	-
5	TP0192	748	206663	207410	-	-
6	TP0200	264	210183	210446	-	-
7	TP0291	834	304706	305539	-	-
8	TP0319	1014	334847	335860	-	1 SNP
9	TP0321–TP0322	2640	336149	338788	-	-
10	TP0323	851	338885	339735	-	1 SNP
11	TP0376	806	400903	401708	-	-
12	TP0377	78	401851	401928	-	-
13	TP0516	1533	556351	557883	-	-
14	TP0519	1277	559215	560491	-	-
15	TP0580	1242	630328	631569	-	-
16	TP0587	183	639620	639802	-	-
17	TP0633	776	691437	692212	-	-
18	TP0683	1047	746899	747945	-	-
19	TP0799–TP0800	2168	866136	868303	-	-
20	TP0806	1397	875808	877204	-	-
21	TP0807	165	877407	877571	-	-
22	TP0808	187	877632	877818	-	-
23	TP0877	998	953710	954707	-	2 SNPs
24	TP0933	2023	1013098	1015120	-	-
25	TP0952	1438	1032341	1033778	-	1 SNP
26	TP0961	1216	1041973	1043188	-	-
27	TP0980	975	1063047	1064021	-	-

ORF – open reading frame; nt – nucleotide; SNP – single nucleotide polymorphism; ^a^as described in [3]; ^b^SNPs identified using CGS in these regions were verified by DDT sequencing.

**Table 3:** DDT sequencing of regions showing different whole genome fingerprint profiles in SS14 strain

Region no.	ORF^a^	Difference from WGF on the gel	Size of sequenced region (nt)^b^	Left coordinate^a^	Right coordinate^a^	Newly found changes	Confirmation of SNPs identified by CGS^c^
1	TP0124–TP0134	insertion	3245 + 1255 insertion	145858	149102	1255 nt insertion, 2 nt deletion	-
			1362	149563	150924	-	-
			252	151648	151899	-	-
			3465	152043	155507	1 nt insertion	2 SNPs

2	TP0135–TP0138	deletion	662	155686	156347	-	(+ 64 nt deletion as in Table 1)
			894	158391	159284	1 nt deletion	

3	TP0433–TP0434	insertion	481 + 419 insertion	461058	461538	insertion of 7 repeats of 60 nt region altogether 14 repetitions, consensus sequence of the repeat CGTGAGGTGGAGGACGYGCCGRRGGTAGTG GAGCCGGCCTCTGRGCRTGARGGAGGGGAG	-

4	TP0468–TP0471	deletion	3571	495308	498878	2 nt deletion + 1 nt insertion + deletion of seven 24 nt repetitions, consensus sequence of the repeat CTCCGCCTCCTTGCGCCGGGCTTC	1 SNP

nt – nucleotide; SNP – single nucleotide polymorphism; ^a^as described in [3]; ^b^regions previously described in Table 1 were excluded; ^c^SNPs identified using CGS in these regions were verified by DDT sequencing.

1674 out of 1731 candidate regions were identified as SNPs in the second sequencing stage but there were 57 regions encompassing 124 oligonucleotide targets where sequence changes could not be determined. These represented possible hypervariable regions with multiple differences from Nichols in the sequencing 29 mers. DNA regions comprising these sites were grouped into 38 larger regions (29–1507 bp), amplified by PCR and DDT sequenced. In 21 of the 38 cases, mostly closely spaced SNPs and/or short insertions or deletions (indels ranging from 1 to 7 nts) were found while no changes were seen in 17 cases (Table 1, column 7), which is in agreement with data obtained by others [12]. DDT sequencing of hypervariable regions suggested by the first phase of CGS identified nucleotide changes in these regions (Table 1, column 7) and also in the vicinity of these regions, where results of the first CGS phase suggested no changes (Table 1, column 8), indicating the need for extension of DDT sequenced regions of at least 100 bp in both directions. Additional short indels were discovered during DDT sequencing of regions identified by WGF (Table 3). Altogether, 2 false positive SNPs (data not shown), 19 false negative SNPs and an additional 16 indels (Tables 1, 3) were found in these DDT sequenced regions (42,344 bp). The overall confirmation of data suggests that repeated regions of the genome are limitations for SNP discovery and almost half of possible hypervariable regions are false positive results.

The accuracy of CGS was determined by comparison to the results of DDT sequencing for 27 regions encompassing 27,141 bp (Table 2). Selection of these regions was focused on possible variable regions in SS14/Nichols hybridization experiments (D. Šmajs, G. M. Weinstock, unpublished results) using a microarray of TPA coding sequences [18] and thus was not completely random. These regions included 5 SNPs and no false positive or false negative SNPs/indels were found. These results indicate an error frequency comparable to or lower than that of high quality finished DDT sequence.

### Assessment of reproducibility of CGS experiments

To test the reproducibility of the method, the genome of TPA SS14 was sequenced twice with the CGS approach, using 2 independent DNA isolations from two subsequent inoculations of rabbit testes (i.e. 4950 and 4951, respectively). When most of the variable genomic regions were excluded from the analysis (CGS cannot identify closely spaced SNPs and/or short indels), CGS discovered 198 SNPs in each DNA preparation. The experiments agreed at 192 SNPs (97%), and 12 SNPs were predicted by only one CGS experiment. Out of these 12 SNPs, 7 were found to be real, as shown by DDT sequencing (data not shown), three loci showed intrastrain heterogeneity in one of the two SS14 DNA isolations, with one allele identical to the Nichols genome sequence and a second allele identical to the base change found by CGS. Two SNPs were predicted falsely, and in both cases the false SNP was located next to a real SNP. The reproducibility of the CGS method is thus likely to be limited by the presence of SNP clusters and influenced by genetically different subpopulations in the test sample.

### Physical mapping of treponemal chromosome

To verify the complete sequence of SS14 strain, to screen for possible discrepancies in cross-reacting repeat regions (*tpr *genes) and insertions of unique sequences, WGF was performed. This physical mapping approach showed the order of the ORFs along the chromosome is identical to Nichols genome and 4 large indel regions were identified. A 64 bp deletion upstream of TP0136 was found by both CGS and WGF methods. Three additional indels were found only by WGF, two insertions (between genes TP0126–TP0127 and within overlapping genes TP0433–TP0434) and one deletion (in TP0470) (Table 3). A deletion in TP0470 and an insertion in TP0433–TP0434 comprised tandem repeats of 24 and 60 bp, respectively. Similar analysis of the Nichols strain revealed length differences in genes TP0433–TP0434 compared to the published sequence [GenBank:AE000520] as described previously [19]. Moreover, intrastrain heterogeneity in the Nichols strain was observed in regions comprising TP0126–TP0127 and upstream of TP0136 with one allele identical to the published sequence. In the Nichols BAC library [20], similar intrastrain heterogeneity was found in the vicinity of gene TP0126. This region comprises a 1255 bp insertion between genes TP0126 and TP0127 in SS14 strain. A similar region was previously described in another syphilitic strain (Chicago, [GenBank:AY587909]) and was found to contain a sequence similar to *tprK *and is believed to be recipient site of the *tprK *conversion [21]. Altogether, three large indels were not detected by CGS. We suggest probable reasons for this fact are (1) the length of the repeats is similar to/longer than oligonucleotides used on the array and (2) sequence changes were found in Nichols DNA when compared to published complete sequence used for mapping array design [GenBank:AE000520].

### Analysis of whole genome interstrain heterogeneity between Nichols and SS14

**Figure 1:**
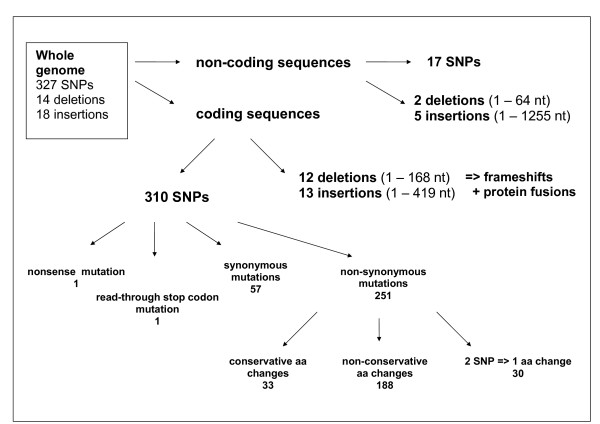
Scheme to identify sequence changes in the SS14 genome.

When results of CGS, WGF and DDT sequencing were combined, 327 SNPs (224 transitions and 103 transversions), 14 deletions and 18 insertions were identified (Fig. 1). Sequence changes of variable regions V1–V7 of TP0897, *tprK*, were not included, because sequences of these regions were found to differ greatly in both length and sequence within the SS14 population, in agreement with investigations published previously [22-24]. Obtained data have been used to compile the sequence of the SS14 genome [GenBank:CP000805]. The GenBank entry contains Ns in the positions of variable regions V1–V7 of *tprK *gene. All discovered sequence changes are listed in Table S1 (See Additional file 1: Supplemental data).

Interstrain sequence heterogeneity discovered between strains Nichols and SS14 included silent mutations, amino acid alterations/indels, gene fusions, and truncations and elongations of open reading frames due to indels. Among the SNPs found by CGS was an adenine to guanine transition in both copies of 23S rDNA in SS14 strain. This sequence change was previously described in association with the SS14 erythromycin resistance [25]. Many discovered indels did not disrupt the open reading frames and represented variable number of nucleotides in homopolymeric tracts (e.g. in TP0012, TP0127), variable number of short motif repeats of 3 and 6 nucleotides (e.g. in TP0136, TP0304, TP0544, TP0668, TP0865), and variable number of longer motif repeats of 60 and 24 nts (TP0433–TP0434, TP0470).

Frameshift mutations and other changes affecting protein length are presented in Table 4. Besides 11 hypothetical proteins (including two possible surface proteins – Tp75 and p83/100), FlaB1 and Tex protein were affected. Sequence changes in four cases led to fusion of ORFs (TP0006 and TP0007 – elongation of Tp75 protein; hypothetical proteins TP0433 and TP0434, TP0597 and TP0598; conserved hypothetical proteins TP0468 and TP0469). Three of these genes (TP0006, TP0470, TP0486) were predicted to code for possible surface protein virulence factors [26]. Moreover, antigen p83/100, hypothetical gene TP0127, conserved hypothetical gene TP0470 and the fused proteins TP0433–TP0434 and TP0468–TP0469 were described to be antigenic in rabbits [27]. Two of the frameshift changes were confirmed to be present in the Nichols strain genomic DNA (TP0486 and TP0598).

**Table 4:** Genes with mutations that significantly affect protein length

ORF^a^	SNPs	Other changes	Result of mutation	Protein function
TP0006	1	read-through stop codon	longer protein (+262 aa), fusion with TP0007	Tp75 protein (possible surface protein)
TP0127	0	1 deletion (2 nt)	frameshift (-103 aa)	hypothetical protein
TP0132	0	1 insertion (1 nt)	frameshift (-44 aa)	hypothetical protein
TP0433–TP0434	1	insertion of tandem repeats	fusion of 2 ORFs (604 aa)	hypothetical proteins (resulting fusion – *arp *protein^c^)
TP0468–TP0469	0	1 insertion (2 nt) 1 deletion (1 nt)	fusion of 2 ORFs (650 aa)	conserved hypothetical proteins
TP0470	0	deletion of 7 tandem repeats (7 × 24 nt)	shorter protein (-56 aa)	conserved hypothetical protein
TP0486	0	1 deletion (1 nt)^b^	frameshift (+9 aa)	antigen, p83/100 (possible surface protein)
TP0598	1	4 insertion (4 nt)^b^	frameshift (+81 aa) fusion with TP0597	hypothetical protein
TP0868	0	1 deletion (7 nt)	frameshift (-168aa)	flagellar filament 34.5 kDa core protein (FlaB1)
TP0924	1	nonsense mutation	shorter protein (-250 aa)	Tex protein
TP1030	7	1 insertion (1 nt)	frameshift (-46 aa)	hypothetical protein

ORF – open reading frame; SNP – single nucleotide polymorphism; aa – amino acid; ^a^as described in [3]; ^b^same sequence change detected in Nichols Houston strain genomic DNA; ^c ^same sequence change described in [19].

SNPs in SS14 were found to be non-uniformly distributed with the number of SNPs per ORF varying from 0 to 49. Hypervariable regions are listed in Table 5 and include ORFs encoding 3 hypothetical proteins, Tpr proteins (TprC, TprL) and outer membrane protein TP0326. TP0326 was predicted to be a virulence factor [26] and was experimentally verified to be an antigen [27]. It is of interest that the most variable region of the genome represents TP0136 (and sequence upstream of this gene) which encodes a protein that is antigenic in both rabbit and human infections [27,28] and was found to serve as fibronectin and laminin binding protein [29].

**Table 5:** ORFs with the highest number of detected SNPs (+ indels)

ORF^a^	SNPs	aa changes	Other changes	Result of mutation	Protein function
TP0117	10	6			Tpr protein C (TprC)
TP0136	49	38	4 deletions (9 nt)	3 aa missing	hypothetical protein^b^
TP0326	12	9			outer membrane protein
TP0515	10	10			conserved hypothetical protein
TP0548	30	21	3 insertions (12 nt)	4 aa inserted	hypothetical protein
TP1031	31	23			Tpr protein L (TprL)

ORF – open reading frame; SNP – single nucleotide polymorphism; aa – amino acid; nt – nucleotide; ^a^as described in [3]; ^b^this protein was described to be fibronectin and laminin protein [29].

The distribution of SNPs in coding and non-coding sequences of SS14 was not significantly different. ORFs represent 92.9% of total genomic sequence; 94.8% of all SNPs were in coding sequences corresponding to 310 SNPs in genes (212 transitions and 98 transversions) and 17 SNPs (5.2%) in intergenic regions (12 transitions, 5 transversions). The frequency of SNPs was different among putative protein classes (Table 6). The highest frequency of SNPs was in hypothetical genes, lowest in housekeeping genes. In addition, housekeeping genes had the lowest number of SNPs altering amino acid sequences indicating conservation of these gene products.

**Table 6:** Distribution of SNPs in different gene function groups and their effects on protein sequences

Putative gene function	whole genome^a^	%	affected ORFs^b^	%	SNPs^c^	%	aa changes^d^	%
Hypothetical	316	30.4	52	38.2	199	64.2	148	67.0
Conserved hypothetical	177	17.0	21	15.4	34	11.0	22	10.0
Metabolic functions	167	16.1	19	14.1	23	7.4	19	8.6
Housekeeping genes	223	21.5	24	17.6	25	8.0	10	4.4
Other function	156	15.0	20	14.7	29	9.4	22	10.0

Total	1039	100	136	100	310	100	221	100

^a^number of genes (ORFs) in the complete genome of TPA Nichols strain [3]; ^b^number of all genes with sequence changes in the genome of SS14 strain; ^c^number of SNP changes identified within ORF groups in the genome, other sequence changes were not included; ^d^amino acid changes caused by SNPs, changes in length of the protein molecule are listed in Table 4.

### Identification of intrastrain variability in TPA population

Because DDT sequencing of some PCR products did not result in an unambiguous sequence, WGS-DDT sequencing of small insert libraries was performed. Analysis of libraries and PCR products revealed multiple (intrastrain) sequence variants in TP0117 (*tprC*), TP0402 (coding for flagellum-specific ATP synthase), TP0620 (*tprI*), TP0621 (*tprJ*), TP0971 (pathogen-specific membrane antigen) and TP1029 (hypothetical protein) and in the intergenic region between *tprI *and *tprJ*. Consensus sequences were mostly identical to the Nichols published sequence, but some positions had minor alternative sequences or *vice versa*. Altogether, intrastrain genetic heterogeneity comprised polymorphisms in 43 nucleotide positions and one polymorphism in a homopolymeric stretch (Table 7).

**Table 7:** Genetic heterogeneity in the SS14 population isolated from rabbit testes

ORF^a^	Genome position^a^	[GenBank:AE000520] sequence	SS14 sequence^b^	position in ORF (Nichols) ^a^	aa change	note
TP0117	135098	G	G or C (5/6)	1600	P534 => A534	
	135107	T	T or C (3/4)	1591	I531 => V531	
	135141	G	G or A (5/2)	1557	no change	
	135144	T	T or C (3/4)	1554	no change	
	135149	C	C or T (5/2)	1549	A517 => T517	
	135220	G	G or A (5/6)	1478	T493 => I493	
	135227	G	G or A (6/6)	1471	P491 => S491	
	135235	G	G or A (2/10)	1463	A488 => V488	
	135239	C	C or T (2/10)	1459	G487 => R487	
	135251	A	A or G (6/6)	1447	Y483 => H483	

TP0402	427435	C	C or T (NA)	401	P134 => L134	
	427737	G	G or T (NA)	703	A235 => S234	

TP0620	671746	T	T or C (9/3)	1142	Q381 => R381	
	671751	T	T or G (19/10)	1137	R379 => G379	
	671753	T	T or C (19/10)	1135	R379 => G379	
	671763	C	C or T (8/4)	1125	no change	
	671982	G	G or C (12/6)	906	S302 => R302	
	672004	C	C or T (12/6)	884	S295 => N295	
	672016	A	G or A (12/6)	872	L291 => P291	
	672025	T	T or C (11/7)	863	N288 => C288	
	672026	T	T or A (11/6)	862	N288 => C288	
	672027	A	A or G (11/6)	861	G287 => D287	
	672028	C	C or T (12/5)	860	G287 => D287	
	672036	G	G or T (11/6)	852	no change	
	672039	A	A or G (NA)	849	P283 => N283	
	672040	G	G or T (NA)	848	P283 => N283	
	672041	G	G or T (12/6)	847	P283 => N283	
	672042	G	G or A (NA)	846	D282 => S282	
	672043	T	T or C (13/6)	845	D282 => S282	
	672044	C	C or T (10/5)	844	D282 => S282	
	672154	G	G or T (7/10)	734	T245 => K245	
	672286	G	G or A (4/12)	602	T201 => M201	

Upstream of TP0620	672916-7	(-)	(-) or C (6/6)	position -30 from TP0620		homopolymeric stretch
	672944	A	A or G (14/6)	position -58 from TP0620		

TP0621	673088	T	T or C (14/4)	2134	I712 => V712	
	673119	G	G or A (14/4)	2103	no change	
	673425	C	C or T (2/8)	1797	no change	
	673428	A	A or G (2/8)	1794	no change	
	673511	A	A or C (6/6)	1711	F571 => V571	
	673545	C	C or T (9/4)	1677	no change	
	673550	A	A or G (10/6)	1672	F558 => L558	
	673554	C	C or T (10/6)	1668	no change	

TP0971	1054447	T	T or C (NA)	301	K101 => E101	

TP1029	1123796	G	G or A (5/6)	15	no change	

ORF – open reading frame; aa – amino acid; NA – not available; ^a^as described in [3]; ^b^numbers in parentheses show sequence reads for each alternative sequence.

## Discussion

Obtaining the complete genome sequence of a second syphilis spirochete (SS14) shows the utility of the CGS strategy for treponemal comparative genomics. This is the first application of this approach to sequence an entire genome. This approach can be used when highly similar genomes are investigated and one genome sequence of closely related organism is known. The CGS strategy represents a rapid (days to weeks) and scalable methodology to sequence multiple syphilitic strains and clinical isolates. In the present study there was a need to further investigate some variable regions, but the directed DDT sequencing required was much less than needed to sequence a whole genome, thus lowering the total cost of obtaining the genome sequence.

There are some of the TPA-specific limitations of this approach to whole genome sequencing. Because the CGS strategy uses genomic DNA as a probe, accuracy is affected by the presence of repeated sequences. Repeat regions hybridize to more than one oligonucleotide on a tiling array resulting in both reduced sensitivity to detect changes, as well as ambiguity in assigning locations for the variants detected. Precautions have to be taken when inspecting *tpr *regions and others (*arp *gene, TP0470) which cross-react based on sequence similarity. Such regions, together with highly variable regions, need to be analyzed by WGF and sequenced by DDT to reveal true nucleotide changes and numbers of repeated regions. Another possible restriction of this methodology arises from the character of the TPA population. Multiple sequence variants in the Nichols strain population were both described previously and identified in this work, and hybridization based sequence changes discovery in these regions is influenced by the ratio between/among different sequence variants in the population. Finally, the accuracy of the genome sequence produced by CGS is dependent on the accuracy of the reference genome sequence. As suggested by two newly revealed frameshifts in Nichols strain sequence, discovered sequence changes have to be verified in Nichols sequence to describe real sequence changes compared to Nichols genome.

The SS14 genome brings a first insight into the whole genome variability within TPA. Both Nichols and SS14 cause infection in rabbits and so are not believed to be attenuated to cause infection in man, thus it is very probable none of the differences may affect the ability of the bacteria to cause the disease. The examples of interstrain heterogeneity and multiple alleles in a population of haploid organisms are candidates for antigenic variation, contingency genes and other types of SSR (short sequence repeats) [30,31]. Changes resulting in significant differences in protein sequences (frameshifts and sequence changes causing protein length shifts) and hypervariable regions affected novel genes, membrane antigens and Tpr proteins. The Tpr protein family includes 12 *Treponema pallidum *repeat proteins, found uniquely in this bacterium and showing sequence similarity to major sheath protein of *Treponema denticola*. 8 out of 12 *tpr *genes (66%) were found to be affected by sequence changes representing a higher proportion than the whole genome rate (13.1%). Positions showing interstrain and intrastrain heterogeneity or both were found in *tpr *genes. Altogether 53 SNPs and 38 intrastrain variable nucleotide positions, with at least one allele identical to the sequence of the Nichols genome, were found in *tpr *genes (V1–V7 regions of *tprK *were excluded from this analysis). Based on the fact that *tpr *genes share a high degree of similarity on the DNA level, we expect differences could be underestimated due to the limitations of the hybridization method for repeated sequences. Multiple alleles of *tpr *genes were described among and within TPA strains [22-24,32,33] and some TPA repeated regions (*tpr *genes, *arp *gene) were used as loci for typing of clinical isolates [34-38]. Newly identified hypervariable regions (Table 5) represent candidate sequences to screen clinical isolates and have potential to be used as typing markers of strains and isolates. In addition, different strains of TPA have already been tested for association with higher risk for neuroinvasion in rabbits [39] and identification of underlying sequence changes will enable prediction of such risks. The identified variation in novel genes suggests other targets besides *tpr *genes could be responsible for antigenic variation in TPA, or without support of further expression and antigenicity data, these could represent pseudogenes.

## Conclusion

The CGS strategy combined with WGF represents a rapid and simply scalable method to assess genome-wide genetic variability within TPA strains and subspecies, which share a very unusual degree of sequence similarity and lack genome rearrangements (as shown in this study). We expect this method to be combined with new sequencing technologies to produce high quality genome sequences to provide important data to design genotyping systems for more intensive strain sampling. Sequence variants could be readily used for molecular typing and identification of SS14 and Nichols strains and, with accumulation of additional data from other TPA genomes, for epidemiologic applications and clinical discrimination between reinfection and reactivation of syphilitic processes. Moreover, the ability to now sequence numerous TPA strains, especially those showing different degrees of virulence, will allow phenotype to be correlated with sequence. This is a significant development for an organism of important public health impact, but for which standard bacterial genetic methods are untenable.

## Methods

### DNA isolation

TPA strains Nichols and SS14 were maintained by rabbit inoculation and purified by Hypaque gradient centrifugation as described previously [40]. Chromosomal DNA was prepared as described previously [3].

### Comparative genome sequencing

100 ng of treponemal genomic DNA was amplified to approximately 100 μg using the REPLI-g kit (Qiagen, Valencia, CA). For each array hybridization, 5 μg of amplified genomic DNA was digested with 0.005 U DNase I in 1× One-Phor-All Buffer (Amersham Pharmacia Biotech, Piscataway, NJ) for 5 min at 37°C, followed by inactivation at 95°C for 15 min. To label the digested DNA fragments, 4 μl 5× Terminal Transferase Buffer (Promega, Madison, WI), 1 nmol Biotin-N6-ddATP, and 25 U Terminal Transferase were added directly to the inactivated digestion mix and incubated at 37°C for 90 min, followed by inactivation at 95°C for 15 min.

Mutation mapping microarrays were designed to map mutations by selecting a 29 mer oligonucleotide every 7 bases for both strands of the complete TPA Nichols genome sequence [3], [GenBank:AE000520]. All 325,138 oligonucleotides were synthesized in parallel as described by others [41,42].

Arrays were hybridized to digested, labeled genomic DNA of Nichols and SS14 strains separately and processed as described in [4] with an additional step after second wash in stringent buffer consisting of staining with a solution containing Cy3-Streptavidin conjugate (Amersham Pharmacia Biotech) for 10 min, and washing again with non-stringent wash buffer. The Cy3 signal was amplified by secondary labeling of the DNA with biotinylated goat anti-streptavidin (Vector Laboratories, Burlingame, CA). The secondary antibody was washed off with non-stringent wash buffer, and arrays were re-stained with the Cy3-Streptavidin solution.

Finally, the stain solution was removed, and arrays were washed in non-stringent wash buffer followed by two 30 sec washes in 0.5 × SSC and a 15 sec wash in 70% EtOH. Arrays were spun dry in a custom centrifuge and stored until scanning.

Microarray scanning, data analysis and sequencing microarray design and procedure were described previously [4]. The second array designed to sequence SS14 strain contained 392,000 oligonucleotides, with 8 oligos per base position (4 for each strand) and 48,600 bases were sequenced in total. Because mutations are sequenced in step two, inclusion of false positives from the mapping arrays does not affect the final data set.

### Dideoxy-terminator sequencing of heterologous SS14 genome regions

After the second sequencing stage of the array analysis, some regions (Table 1) of the SS14 genome showed clear differences but SNPs could not be clearly identified. These regions were sequenced by DDT sequencing. Coordinates of these regions were extended with at least 150 bp in both directions and amplified with Taq-polymerase using oligonucleotide primers designed with Primer3 software [43]. The resulting PCR products were purified using QIAquick PCR purification Kit (Qiagen) and DDT sequenced using the original amplification primers and internal primers where applicable. Due to sequence similarity between *tpr *(*Treponema pallidum *repeat) genes, 3 of the heterologous regions (comprising genes TP0620–TP0621, TP0896–TP0898, TP1029–TP1030) were XL PCR amplified using primers annealing to unique regions in the vicinity of the desired sequence. XL PCR products were purified and mechanically sheared to fragments 500 – 1000 bp in length. These fragments were cloned into the pUC18 vector resulting in small insert libraries and recombinant plasmids isolated from at least 48 colonies were DDT sequenced to multiple coverage using pUC18 primers. All sequence reads were analyzed using Lasergene software (DNASTAR, Inc., Madison, WI).

### Whole genome fingerprinting

Whole genome fingerprinting was performed as described previously [44]. The chromosomal DNA was amplified in 102 *Treponema pallidum *interval (TPI) regions with median length of 12,204.5 bp (ranging from 1,778 to 24,758 bp) using the GeneAmp^® ^XL PCR Kit (Applied Biosystems, Foster City, CA). The primer pairs for these amplifications are listed in Table S2 (See additional file 1: Supplemental data). Each PCR product was digested with *Bam*H I, *Eco*R I and *Hin*d III (New England Biolabs, Ipswich, MA) or their combinations. To asses the possible presence of indels in restriction fragments ≥ 4 kb, additional digestions using *Acc *I, *Cla *I, *EcoR *V, *Kpn *I, *Mlu *I, *Nco *I, *Nhe *I, *Rsr *II, *Sac *I, *Spe *I, *Xba *I or *Xho *I were performed as needed. The resulting fingerprints of TPA Nichols and SS14 strains were compared.

### Nucleotide sequence accession number

The complete sequence of TPA SS14 strain was deposited in the GenBank under the accession number CP000805.

## Authors' contributions

GMW designed the study. PM performed genome sequence analysis, finishing using DDT sequencing and wrote the manuscript. MS and ES performed WGF analysis. JEN, JS, TAR, MNM, TJA composed the CGS technique team and analyzed hybridization data. JFP contributed to SNP and proteome analysis. DS, TP, SJN and GMW provided critical expertise of the manuscript. All authors read and approved the final manuscript.

## Supplementary Material

Additional file 1 Supplemental material consists of two tables containing list of all identified sequence changes in TPA SS14 genome compared to [GenBank:AE000520] (Table S1) and list of primers used for WGF analysis (Table S2). Click here for file
