# Supplementary material for: Complete genome sequence of Treponema pallidum ssp. pallidum strain SS14 determined with oligonucleotide arrays
Source: BMC Microbiol. 2008 May 15;8:76. doi: 10.1186/1471-2180-8-76 (PMC2408589; doi:10.1186/1471-2180-8-76)
Supplement: Additional file 1 — Supplemental material consists of two tables containing list of all identified sequence changes in TPA SS14 genome compared to [GenBank:AE000520] (Table S1) and list of primers used for WGF analysis (Table S2). [file 1471-2180-8-76-S1.pdf]

Table S1. List of all identified sequence changes in TPA SS14 genome.

Coordinates in AE000520	Sequence change	AE000520	SS14	ORF in AE000520
6442	SNP	G	a	TP0005
7179	SNP	T	c	TP0006
9983	SNP	A	g	TP0008-TP0010
12476-12478	3 nt deletion	GGG	(-)	TP0012
20493	SNP	G	a	TP0018
22506	SNP	C	g	TP0020
41126	SNP	C	t	TP0033
47521	SNP	C	t	TP0039-TP0040
59887	SNP	T	c	TP0051
73150	SNP	A	g	TP0067
79957	SNP	T	c	TP0073
83983	SNP	C	g	TP0076
85603	SNP	G	a	TP0077
87999	SNP	A	c	TP0079
94064	SNP	C	t	TP0083
94889	SNP	C	a	TP0085
132270	SNP	C	t	TP0115
134922	SNP	G	a	TP0117
134946	SNP	T	g	TP0117
134954	SNP	A	c	TP0117
134963	SNP	C	g	TP0117
134964	SNP	G	a	TP0117
134966	SNP	C	t	TP0117
134970	SNP	G	a	TP0117
135271	SNP	C	a	TP0117
135272	SNP	G	a	TP0117
135275	SNP	T	c	TP0117
148104	SNP	A	c	TP0126
148526-148527	1255 nt insertion	(-)	see 148524-149778 in CP000805	TP0126-TP0127
148944-148945	2 nt deletion	GG	(-)	TP0127
149342	SNP	C	g	TP0127-TP0128
149344	SNP	G	c	TP0127-TP0128
149349-149350	1 nt insertion	(-)	c	TP0127-TP0128
149350-149351	5 nt insertion	(-)	ggcca	TP0127-TP0128
151098	SNP	T	c	TP0130-TP0131
151435	SNP	G	a	TP0131
151439	SNP	C	t	TP0131
151471	SNP	C	a	TP0131
151472	SNP	G	a	TP0131
151475	SNP	T	c	TP0131
153123-153124	1 nt insertion	(-)	g	TP0132
154598	SNP	C	t	TP0134
155507	SNP	G	a	TP0135
156488-156551	64 nt deletion	see AE000520	(-)	TP0135-TP0136
157042	SNP	G	a	TP0136
157120	SNP	A	c	TP0136

157123	SNP	C	a	TP0136
157124	SNP	G	t	TP0136
157183	SNP	G	t	TP0136
157185	1 nt deletion	A	(-)	TP0136
157187	SNP	C	g	TP0136
157188	1 nt deletion	T	(-)	TP0136
157191	1 nt deletion	A	(-)	TP0136
157195	SNP	C	g	TP0136
157197	SNP	G	a	TP0136
157203	SNP	A	g	TP0136
157212	SNP	C	g	TP0136
157213	SNP	C	g	TP0136
157233	SNP	C	a	TP0136
157235	SNP	G	t	TP0136
157264	SNP	A	c	TP0136
157275	SNP	C	a	TP0136
157297	SNP	A	g	TP0136
157321	SNP	G	c	TP0136
157343	SNP	G	c	TP0136
157381	SNP	G	c	TP0136
157393	SNP	A	g	TP0136
157417	SNP	G	c	TP0136
157439	SNP	G	c	TP0136
157467	SNP	G	a	TP0136
157468	SNP	T	a	TP0136
157473	SNP	A	g	TP0136
157480	SNP	A	g	TP0136
157498	SNP	T	g	TP0136
157570	SNP	A	c	TP0136
157577-157582	6 nt deletion	CGGTGG	(-)	TP0136
157623	SNP	G	c	TP0136
157636	SNP	C	a	TP0136
157645	SNP	C	t	TP0136
157731	SNP	G	a	TP0136
157733	SNP	C	t	TP0136
157734	SNP	A	g	TP0136
157764	SNP	C	t	TP0136
157765	SNP	A	c	TP0136
157825	SNP	C	a	TP0136
157933	SNP	A	g	TP0136
157996	SNP	G	a	TP0136
158005	SNP	G	a	TP0136
158146	SNP	A	g	TP0136
158157	SNP	T	a	TP0136
158161	SNP	G	a	TP0136
158166	SNP	G	a	TP0136
158167	SNP	G	c	TP0136
158169	SNP	A	g	TP0136
158172	SNP	A	g	TP0136
158223	SNP	A	t	TP0136

158225	SNP	C	g	TP0136
158348	SNP	C	g	TP0137
158396	1 nt deletion	T	(-)	TP0137-TP0138
170364	SNP	A	g	TP0147
196255	SNP	A	g	TP0178
196890	SNP	G	a	TP0179
196995	SNP	T	g	TP0179
197036	SNP	C	t	TP0179
197119	SNP	T	c	TP0179
197184	SNP	A	g	TP0179
197202	SNP	T	c	TP0179
197246	SNP	C	t	TP0179
197278	SNP	C	t	TP0179
234049	SNP	A	g	TP0225-TP0226
236591	SNP	T	g	TP0227-TP0228
238914	SNP	T	c	TP0230
241511	SNP	G	a	TP0233
263726	SNP	T	c	TP0252
269952	SNP	T	g	TP0258
269953	SNP	T	c	TP0258
277584	SNP	G	a	TP0265
282493	SNP	A	g	TP0266-TP0267
300962	SNP	G	a	TP0287
310927	SNP	T	a	TP0297
310929	SNP	G	t	TP0297
313326	SNP	A	g	TP0300
317931	SNP	C	a	TP0304
319010-319012	3 nt deletion	CTG	(-)	TP0304
319533	SNP	C	t	TP0304
319675	SNP	T	c	TP0304
326267	SNP	G	a	TP0309
326443	SNP	T	c	TP0310
326507	SNP	T	c	TP0310
333324	SNP	G	a	TP0317
335063	SNP	A	c	TP0319
345868	SNP	G	a	TP0326
345872	SNP	T	g	TP0326
345875	SNP	T	a	TP0326
345877	SNP	C	a	TP0326
345902	SNP	A	g	TP0326
345903	SNP	C	t	TP0326
345909	SNP	G	a	TP0326
345916	SNP	G	c	TP0326
345924	SNP	G	a	TP0326
346242	SNP	T	g	TP0326
346663	SNP	A	c	TP0326
346796	SNP	A	g	TP0326
352194	SNP	T	c	TP0330
353805	SNP	G	a	TP0330-TP0331
368892	SNP	A	g	TP0344

371391	SNP	G	a	TP0346
371741	SNP	A	g	TP0346
407589	SNP	G	a	TP0381
412101	SNP	A	g	TP0387
413671	SNP	G	a	TP0389
420603	SNP	T	c	TP0394
434694	SNP	A	g	TP0408
458814	SNP	T	c	TP0430
459288	SNP	A	g	TP0431
461079-461507	different sequence (+419 nt insertion compared to AE000520)	see AE000520	see 462259-463106 in CP000805 consensus sequence of the repeat CGTGAGGTGGAGGACGYGCC GRRGGTAGTGGAGCCGGCCT CTGRGCRTGARGGAGGGGAG	TP0433+TP0434
461844	SNP	T	c	TP0434
469810	SNP	A	g	TP0443
476053	SNP	G	a	TP0449
491190	SNP	A	g	TP0462
491191	SNP	A	g	TP0462
491268	SNP	A	g	TP0462
491287	SNP	A	c	TP0462
491313	SNP	G	a	TP0462
491342	SNP	A	c	TP0462
491413	SNP	G	a	TP0462
495738-495739	2 nt insertion	(-)	gc	TP0468+TP0469
495741	1 nt deletion	G	(-)	TP0469
497265-497432	168 nt deletion	see AE000520 consensus sequence of the repeat CTCCGCCTCCTTGCGCCGGGCTTC	(-)	TP0470
498589	SNP	A	c	TP0471
501839	SNP	T	c	TP0473
510766	SNP	G	a	TP0481
516171	SNP	C	t	TP0485
517501-517502	1 nt insertion	(-)	c	TP0485-TP0486
517597	1 nt deletion	T	(-)	TP0486
519506	SNP	C	a	TP0487
519566	SNP	G	a	TP0487
520170	SNP	C	t	TP0487
521446	SNP	T	a	TP0488
521457	SNP	G	a	TP0488
522138	SNP	T	c	TP0488
522528	SNP	G	a	TP0488
527228	SNP	A	g	TP0492
528553	SNP	C	t	TP0493
535985	SNP	C	t	TP0500
536260	SNP	G	t	TP0501
542841	SNP	G	a	TP0507
548745	SNP	T	c	TP0512
554286	SNP	G	a	TP0515
554689	SNP	A	g	TP0515
555234	SNP	A	g	TP0515
556012	SNP	C	t	TP0515

556017	SNP	C	a	TP0515
556026	SNP	C	a	TP0515
556033	SNP	A	g	TP0515
556084	SNP	A	g	TP0515
556182	SNP	T	c	TP0515
556185	SNP	T	c	TP0515
562957	SNP	A	g	TP0521
572977	SNP	A	g	TP0529
575340	SNP	T	c	TP0531-TP0533
586237-586238	6 nt insertion	(-)	ttcctg	TP0544
590699	SNP	T	c	TP0547
591023	SNP	T	c	TP0547
591562	SNP	A	c	TP0547-TP0548
591830-591831	3 nt insertion	(-)	tgg	TP0548
591845	SNP	C	g	TP0548
591847	SNP	G	a	TP0548
591850	SNP	A	g	TP0548
591851	SNP	G	a	TP0548
591853	SNP	G	a	TP0548
591861	SNP	G	c	TP0548
591972	SNP	T	c	TP0548
592095	SNP	C	a	TP0548
592141-592142	4 nt insertion	(-)	acgg	TP0548
592142-592143	5 nt insertion	(-)	atgat	TP0548
592147	SNP	G	a	TP0548
592151	SNP	A	g	TP0548
592156	SNP	G	a	TP0548
592159	SNP	G	a	TP0548
592162	SNP	A	g	TP0548
592163	SNP	A	g	TP0548
592440	SNP	C	g	TP0548
592441	SNP	A	g	TP0548
592442	SNP	G	a	TP0548
592444	SNP	T	a	TP0548
592447	SNP	C	g	TP0548
592449	SNP	A	c	TP0548
592453	SNP	G	a	TP0548
592547	SNP	G	a	TP0548
592552	SNP	T	c	TP0548
592553	SNP	C	t	TP0548
592593	SNP	A	t	TP0548
592596	SNP	C	g	TP0548
592624	SNP	G	a	TP0548
592630	SNP	G	a	TP0548
592631	SNP	G	a	TP0548
592980	SNP	C	t	TP0548
600625	SNP	C	t	TP0554
604573	SNP	T	g	TP0558
604579	SNP	C	t	TP0558
604637	SNP	T	c	TP0558

604960	SNP	T	c	TP0558
604999	SNP	C	t	TP0558
605347	SNP	A	g	TP0559
605577	SNP	C	t	TP0559
606845	SNP	C	t	TP0560
618367	SNP	T	c	TP0569
620599	SNP	T	c	TP0571
622389	SNP	G	t	TP0574
628411	SNP	G	a	TP0577
633825	SNP	G	t	TP0583
634785	SNP	G	a	TP0584
641300	SNP	T	c	TP0591
646557	SNP	A	g	TP0594
649125-649126	1 nt insertion	(-)	g	TP0598
649205-649206	1 nt insertion	(-)	a	TP0598
649208-649209	1 nt insertion	(-)	a	TP0598
649225-649226	1 nt insertion	(-)	a	TP0598
650613	SNP	T	c	TP0598
661596	SNP	A	c	TP0610
665741	SNP	C	t	TP0612
672175	SNP	C	a	TP0620
672176	SNP	G	c	TP0620
672182	SNP	G	t	TP0620
672186	SNP	C	g	TP0620
688824	SNP	G	a	TP0630
701540	SNP	T	c	TP0640
706966	SNP	G	a	TP0644
729336	SNP	C	t	TP0667
730296-730301	6 nt deletion	ACAGCA	(-)	TP0668
748158	SNP	A	g	TP0683-TP0684
766386	SNP	G	a	TP0699
789385	SNP	G	t	TP0721
798332	SNP	A	g	TP0732-TP0733
802220	SNP	A	g	TP0736
803125	SNP	G	a	TP0737
810637	SNP	A	g	TP0746
814853	SNP	C	t	TP0748
820357	SNP	T	g	TP0756
836111	SNP	A	g	TP0770
857217	SNP	A	g	TP0790
858808	SNP	G	a	TP0792
859962	SNP	G	t	TP0793
872547	SNP	C	t	TP0804
888551	SNP	C	t	TP0820
914612	SNP	T	c	TP0841
928111	SNP	C	t	TP0854
934573	SNP	G	a	TP0858
935544	SNP	G	a	TP0858
935575	SNP	C	t	TP0858
943688	SNP	C	t	TP0865

944088-944089	3 nt insertion	(-)	gtt	TP0865
944226	SNP	C	t	TP0865
944866-944867	1 nt insertion	(-)	c	TP0865-TP0866
946867	SNP	A	g	TP0867
947060	SNP	T	c	TP0867-TP0868
947437-947443	7 nt deletion	CGGCAAA	(-)	TP0868
949989	SNP	G	a	TP0872
954310	SNP	T	c	TP0877
954674	SNP	C	a	TP0877
974232	SNP	T	c	TP0896
974234	SNP	G	a	TP0896
974463-974543	tprK variable region V7	see AE000520	81 Ns	TP0897
974619-974669	tprK variable region V6	see AE000520	51 Ns	TP0897
974751-974834	tprK variable region V5	see AE000520	84 Ns	TP0897
974877-974927	tprK variable region V4	see AE000520	51 Ns	TP0897
974958-975002	tprK variable region V3	see AE000520	45 Ns	TP0897
975004	SNP	T	c	TP0897
975099-975164	tprK variable region V2	see AE000520	66 Ns	TP0897
975582-975623	tprK variable region V1	see AE000520	42 Ns	TP0897
976292	SNP	A	g	TP0898
976850	SNP	T	c	TP0898
991155	SNP	A	g	TP0912
991156	SNP	A	c	TP0912
992749	SNP	A	g	TP0915
1004582	SNP	C	a	TP0924
1033494	SNP	C	t	TP0952
1040937	SNP	T	c	TP0958
1047926	SNP	G	c	TP0966
1048604	SNP	G	a	TP0966
1048823	SNP	G	a	TP0966
1051304	SNP	C	t	TP0968
1057842	SNP	T	c	TP0973
1058010	SNP	C	t	TP0973-TP0974
1062062	SNP	T	g	TP0978
1079978	SNP	G	a	TP0995
1100588	SNP	A	g	TP1008-TP1009
1102291	SNP	T	g	TP1010-TP1011
1122613	SNP	C	t	TP1028
1123760	SNP	C	t	TP1029
1123935	SNP	A	g	TP1030
1124003-1124004	1 nt insertion	(-)	c	TP1030
1124255	SNP	G	c	TP1030
1124302	SNP	C	t	TP1030
1124380	SNP	C	g	TP1031
1124406	SNP	G	a	TP1031
1125098	SNP	G	a	TP1031
1125107	SNP	G	c	TP1031

1125108	SNP	C	a	TP1031
1125126	SNP	T	c	TP1031
1125131	SNP	C	t	TP1031
1125149	SNP	T	c	TP1031
1125161	SNP	C	a	TP1031
1125162	SNP	A	c	TP1031
1125164	SNP	A	g	TP1031
1125167	SNP	A	g	TP1031
1125171	SNP	T	a	TP1031
1125173	SNP	G	c	TP1031
1125191	SNP	A	g	TP1031
1125197	SNP	C	g	TP1031
1125201	SNP	C	t	TP1031
1125206	SNP	G	a	TP1031
1125224	SNP	A	g	TP1031
1125225	SNP	A	c	TP1031
1125381	SNP	A	g	TP1031
1125401	SNP	C	t	TP1031
1125402	SNP	T	g	TP1031
1125540	SNP	A	g	TP1031
1125562	SNP	G	a	TP1031
1125563	SNP	A	g	TP1031
1125564	SNP	A	g	TP1031
1125567	SNP	C	a	TP1031
1125568	SNP	T	g	TP1031
1125579	SNP	G	a	TP1031
1125580	SNP	G	c	TP1031

Table S2. List of primer pairs used for WGF analysis in TPA SS14.

Region no.	TPI interval	Forward primer	Reverse primer	Region length (bp)
1	TPI-1	CGGCTGTATTTCGTTACTGTCTTGA	GGGTTTGGTAGTGACCTCTCTATGGT	11757
2	TPI-2	TCTCCCGAACGAGTTTCTAGTCTG	AACTTAACGCTGCACATAGTACACACA	19610
3	TPI-3	TGTTTTACCAGGTTGCAGGGAC	CGGAAAGAAATCCACACGGAG	12126
4	TPI-4A	CTCCGTGTGGATTTCTTTCCG	GTCACGCTTCAAAATGGCGCC	6348
5	TPI-4B	CAGGCGCCATTTTGAAGCGTGACATTT	GCGTTTGCATGGGTAAGACTAACTT	7705
6	TPI-5A	CACCAATGAGAGAACGCATCAGC	GGTGGTACCGGCCAAACAGCACG	12087
7	TPI-5B	CGCTACCACACCCTATCATACCG	TGAAGCGCTTGTTGCAGCAACCG	12643
8	TPI-6	TCATCAAAAAGAAGTACGCTGTAGGG	CTCCTCCGCGTACTCCAAGAC	14827
9	TPI-7A	GCGCCTGATGAAAGTTGACC	CACGCGCTTCCAAATAGTCC	10792
10	TPI-7B	CGTGCACATGCACGCCAAACGCG	GTAAGAGCGCTTCTACATCCTCACA	11998
11	TPI-8	GATTCTGCCGTTTTGGTAACTACTTG	TAACTCATGCCCAACAGTAGCTTG	5053
12	TPI-9	GGATTGCTTTCTGTGTTTGAGACC	TTCACAGATTAACGGCATTGGAC	5351
13	TPI-10	ACCGCGGAACGAATCAAGTAG	CAATACCCCATCTCTCCGAGC	11261
14	TPI-11	TTATCAGCCTGAATCGTATGTCCC	ATGTGCAAGTACTCCAACCTCTCG	12733
15	TPI-12	GGTTGGAGTACTTGCACATGTGG	TCATACGTTTTTTCGCTCCACAC	9838
16	TPI-13	TCTGCCTTACAGGTGACGTCCT	CTCCAATACACGAAACTATCTTGCG	4761
17	TPI-13B	GTGTGGAGCGAAAAAAACGTAT	CTTGGCCACAATCGTGATTTG	3647
18	TPI-14A	TGTATCTGCGTTCCTAATACACC	CGAATCGCTCGAATGCGTAGGTG	13048
19	TPI-14B	AAGCCGACACAGCTCACTCC	CAACACACGCCTTTCAGCAG	13877
20	TPI-15	TGTATGTCGTGCAGTTCTAGCCC	CCTGCGCCTTTAAACTTAGGAGAG	22357
21	TPI-16A	TCAAGCGTGAAAGATAGGGGTG	CGCCGCCGCACACTTTCTTCTCCT	12376
22	TPI-16B	TGGGCAATGGAGGGGTGGTATGTCG	CGCCTTTGTACCCGAGCTTAC	12363
23	TPI-17A	GAAGCAGAATGCTGTCTCTCGTG	TTAACTGCGCCAGCGACAAG	14237
24	TPI-17B	ACAAGGTGCGTGTGGCAGTC	ATGGCGTACGTAGGTCCGTGT	13508
25	TPI-18	GCGCAGTAAAAGAGGGACGAC	GATCGCTTCGTACTTCCCTAGCTT	4817
26	TPI-19	GCGCAGTAAAAGAGGGACGAC	CATCATCACGTACAAACCATGAGG	6413
27	TPI-20	CCTCTCGCGTTATTGAGGCTC	TACTAAGCTTCCTCATCATCGTCTCC	8493
28	TPI-21	TCCTTTCCGATGAAATGGACCT	GTTTGGGCATCGGTCTAGAGG	20429
29	TPI-21B	CAGTCTGTGGGGGATGACTAGGT	CAGGCGCACATCTCCTTCTTAG	15070
30	TPI-22	TCCTTTCCGATGAAATGGACCT	CAGCGCGGCAGTTCTTATACTTAG	6995
31	TPI-23	GTTTCGAACTGGAAAATACGTGTGTAC	GACAAGCGAGCGTGTAGGAGTC	18499
32	TPI-24A	GACTCCTACTCGCTCGCTTGTC	ACTGCGGTGCCGCTCCACTGG	8048
33	TPI-24B	CCAGTGGAGCGGCACCGCAGTTAC	CCGTGTGGTTCAAATCCAAACT	9020
34	TPI-25A	TTCCGAGCCATATCTGCGTACT	CGTTTAATGTTCTGCGGCCGGTGTTTG	5764
35	TPI-25B1	GGGCGCCTTCCGGCAGGACTCT	GGAACCACTATCTCCTTCGAGACAA	5568
36	TPI-25B2	CAGGATTACGGCTGGGTGAA	GGAACCACTATCTCCTTCGAGACAA	5865
37	TPI-26	CGTCTCAATGGTCAGGACGTG	GCACTATGTATCTGGTCAGGAGCAC	14792
38	TPI-27	CCTTGTTATCATGGACGAAGTAGGAC	GTAATGCTTGCTGCGACGATTAG	16681
39	TPI-28	TGAAATGTTACGCGCTAGAGGG	GCAACCTCGTAGCCTTCACTTGT	23437
40	TPI-29	CAGATTGGTCAAATCTTGACAAGTGA	GATCAAGCTCAACCTCCTGGG	19444
41	TPI-30	GTACAATCTCGATGAAAAGGGGC	ACATGGACCGCCTTCCTCATA	20710
42	TPI-31A	TATACTGTTTTGGTAGATGGGGACGA	TTAGCGCGAGTGAGAAATCCCGACTGC	10405
43	TPI-31B	GTGCGTCAGGCGTTGTTGTTGGTTTTG	ACTCAAACACCAACCCCCTCTC	9902
44	TPI-32A	CACTTTCCTCCGAGGACGTGT	GCGCCGCACGACGATCAAATGA	10655
45	TPI-32B	TGTGCCCGGCTATTGGGAGGTAGACAT	CTGCGCAACTGTTCCTCCATA	11107
46	TPI-33A	TGATGTGATGCAAAGGGTTGAC	GCCATCACGTATGTGCGATGGC	11795

47	TPI-33B	TACATCTGTTGTTCCTGGTAGTCC	GAGCTAAGTTCACAGGGCATTTGC	11993
48	TPI-34	AAATAGAGGCAATCCGAGGGG	GCGTCCTTCTGAATCTCTCGTG	13054
49	TPI-35	CAGTGTGCTTTGTTTTTGGTAGGG	GCGTCCTTCTGAATCTCTCGTG	10899
50	TPI-36	GCAGCGCTTCAGGTGATAGTAGAG	CTGCAAGCGAATCACTAGGTGTC	6931
51	TPI-37	CCTGCAGGGTACGTAAGTAGAGGAC	CGCTTTGATGAGGGAATAGAAGAC	7040
52	TPI-38	TGACCCAATGAAATGACCCTTC	CAGCATGGTCAGAAGCAATAGGTAG	22457
53	TPI-39	GGCAGGGTTACATGCTTGTCTC	TTTCTCACGGTGTCCCCTTATG	20815
54	TPI-40	TTGGGTATCGCTTACACTCGGA	ACGTACGGGAATGAGGATGTACAC	16322
55	TPI-41	TCACCACCTTTGACAGTACCCC	GTGAGTCATGGATCTGCGAGTG	20309
56	TPI-42A	GTTCTGCCATGCGCACCGCCATACG	TGGCGGCTACCTTCGCAGAACTTGC	10127
57	TPI-42B	TGCAAAGCAAATATCGCAATGAGC	ACATCATTGCAAGCATTATCCG	11204
58	TPI-43	GGATGGAAAATTCGTTGTACCCT	CAAGCGCAGTAAAATCCCTCAG	20309
59	TPI-44	GCGTTTTTCCTGTTCTCCCTCT	GGACGCCTCACGTACGTAGATTAC	18747
60	TPI-45	CGCATATGCAGCAGGACAAGT	AGCGGTAATGATCGGTGTGTG	10988
61	TPI-46	CCTTTGCAGAAGAAATCGCATAG	AGTGCATAACGCGCTCTAGCA	7000
62	TPI-47	GCATAACCGCGTGCTATACGA	AGCGCCTCTACATCCTCCATG	2003
63	TPI-48	AGATCGTTCTTGCTATCTCCAATCA	CCGTAGCGCTTTGCTAAGGAC	11460
64	TPI-49	GCCACTCACCTGAAGATTGGAC	TCGAAGCGAACCATTTTCCTAAT	10216
65	TPI-50	TGGTTCGCTTCGATTATCATAACTTAA	GTTTGCGTACTGTACACCGAGTGA	20366
66	TPI-51A	AGTCATTGCGTTTTTCTCGGG	GCGGCGGTGAATGAAATGCTCGAC	13509
67	TPI-52	AGTCATTGCGTTTTTCTCGGG	GGTCAGATAGGACAAGGGGTCAC	15156
68	TPI-53	CATGACCGTTTCAAAGGGCTC	CAGTTTGCTCTACCTGCGTCCT	16343
69	TPI-54A	CGCCCATCAAGACAATCTCC	CTCGCTTGGAGAAACGCACT	14555
70	TPI-54B	CTGCCTATCTTCCCGCACTG	CATGCTCCTTCCCTTTGTGC	11560
71	TPI-55	GGGTTGTTTTGCCAGACGACT	GTATTACCGCAGTACGCTCAGTCC	12697
72	TPI-56	CCATCGTTACCGTTTTCTCTAGCA	TGAAAATTCAGTAATGGAAGGGGTC	17461
73	TPI-57A	CCGTCACTCATACAATTCCTCCTC	CAGAAGATGAGGGGGTGACG	11814
74	TPI-57B	TGCCCATTTCATCAGCAACC	CCGGTCATTGATATGACGAGTGT	11349
75	TPI-58	GACCTTTACCACAAACCTTCTCGA	CGCTGCACTGTACCACAAGTAGAC	12343
76	TPI-59	CGTTTACCATTCTCGTCGAGGTACT	AATCAAGGCGATGTTCTTGGG	16482
77	TPI-60	GGAAAAAAAAGCTACCTCACTCGG	AACCACATGGATGCAGTTACAGTGT	21000
78	TPI-61	TCAAAGAACGCTCGGACACAC	GGTAGAACTATTGTCTGCGTCTCACAT	17465
79	TPI-62A	GCTCTGCAGGGCTCGCTACATGG	CGACACGCCGACGCGTATGCAGTG	13658
80	TPI-62B	GCGCCTCCTGGCATAGGGCAACC	CTCACCTTTTATGTCGGTGTAGATCC	13677
81	TPI-63	GCCAGTATTGGAAAAAGTCATAGGG	CTCGCCACTCACCTTACACGA	14802
82	TPI-64	TTTAATGCGGATCATGAGGTCC	GGTGCATTTTGCAGAGTCAAGAG	11748
83	TPI-65	CTCAATCCACCAGTGTGTGGG	AAGGCTGCAGATGGTCCAAAG	12396
84	TPI-66A	AATAGCCACGGGTCACTAGCATT	AACGCCGGGGATAAGGTCGGTGATAGA	12037
85	TPI-66B	TTGGGTGCCTACCAGAATCG	CGGGAACGATAGACGCTGAC	13776
86	TPI-67	TGTGTGAGATTCAAATCCCAAGG	TTCCCTGGTGTGCGTATAGCA	23536
87	TPI-68	CAGCAAGGGAAAATCCTCCAC	AGATCCAGTAATCCGTATCACACGAG	23288
88	TPI-69	CCATCAGCACAAAGGGTGTGT	CAGAGTGGACCCTCGTGTTCTTAG	24758
89	TPI-70	CCATACACATCCATGTACTCGCAC	AGTATTTTGCGCGTAGGCGTC	14302
90	TPI-70A	CCATACACATCCATGTACTCGCAC	AACGCGGTGCATCGGTCTGTCCAC	7552
91	TPI-70B	AAGCCGGGGTCTCCATCGTCCATCTT	AGTATTTTGCGCGTAGGCGTC	7252
92	TPI-71	GCCATAGGAAACCGTAAGACCG	CGAGGATTTTGAGCATGCCTAG	12283
93	TPI-72	CGTAAGTGATCGCATGTCCCTC	CGTTTGAGCACGAAGGGTACC	16289
94	TPI-73	CACGCTGGTAATTATGGGAGAGAC	TTGACCTAAGCAAGGGCACCT	10361
95	TPI-74A	GCGGTCAACAAGGTAGGAAGC	CGCGCAGAAAAAGTGGAATG	13699
96	TPI-74B	GCGGCATCGCAAGATATACG	CACAGCGAGTATTTAAGGGTGGG	13108

97	TPI-75	TTCTTGCCTTAATCTGTCCGAGC	CACCTATCTTTGTGTGGTAGGGGA	14582
98	TPI-76	ATACCATTCTTCCAGCCCCGT	CACCCTGACGGTGAGTCACTCA	1778
99	TPI-77	GGTTCAATCCCAGACGGACTG	CCCCGTTGTTTACGTAGGAGGT	2823
100	TPI-78	TACGTATACCTTCCCGCGCTC	GGACGTGCAGTTCCATCCTAAG	6494
101	TPI-79	GTAACCGAATTTTGGGCACAAGT	GGACGTGCAGTTCCATCCTAAG	5041
102	TPI-80	CTCAATGTGCTGTTTGTAGTCGGA	ACACTCCTCCTGCCTTGGAGAA	7949
